# Meeting report of the International Workshop on Quantitative Biology 2012: mesoscopic and microscopic worlds meet

**DOI:** 10.3389/fphys.2012.00479

**Published:** 2013-01-11

**Authors:** Viji M. Draviam, Akira Funahashi, Noriko Hiroi, Akatsuki Kimura, Tetsuya J. Kobayashi

**Affiliations:** ^1^Department of Genetics, University of CambridgeCambridge, UK; ^2^Department of Biosciences and Informatics, Keio UniversityYokohama, Japan; ^3^Cell Architecture Laboratory, National Institute of GeneticsMishima, Japan; ^4^Institute of Industrial Science, University of TokyoTokyo, Japan

## Introduction

Over the past decade or two biology has become increasingly quantitative and concepts from mathematical and physical sciences have in turn increasingly influenced biology (Knight, 2002; May, 2004; Endy, 2005; Chuang et al., 2010). As a result of these advances in quantitative biology, new biological phenomena at mesoscopic and microscopic scale have been unraveled and techniques to address long-standing fundamental questions have been developed. Yet quantitative biology as a field is reliant on concepts borrowed from engineering and physics and hence is critically dependent on close interaction between theoretical and experimental biologists. The report reviews the highlights of the interactive workshop, and proposes the long-term benefits of such small-scale cross-disciplinary workshops.

## Meeting report

The International Workshop on Quantitative Biology (IWQB2012) organized in conjunction with the 5th annual meeting of the Japanese Society for Quantitative Biology (Q-BioJP) was held on November 22nd, 2012 in Tokyo.

An opening remark by Prof. Yasuyuki Sakai, Institute of Industrial Science, University of Tokyo, highlighted the scientific importance of quantitative biology for mechanistic understanding of living processes. Prof. Sakai emphasized the need to extend the technical and conceptual knowledge gained into applications area for the benefit of human medicine.

The day's talks were kicked off with a session on mesoscopic structures and their regulation. Prof. Madan Rao (National Center for Biological Sciences, India) opened this session with captivating description of the physical principles that govern the functional organization and dynamics of molecules on the cell surface. Dr. Chun-Biu Li (Hokkaido University, Japan) described his novel approach on how one could develop objective kinetic scheme to identify network properties that are entirely supported by data in a parameter-free manner. Dr. Ziya Kalay (Kyoto University, Japan) ended the session with an elegant description of a basic theoretical framework on molecules' movement on membrane surfaces that could be used to predict reaction bursts.

A live relay of how automated fermentation systems yield data on membrane and metabolite dynamics by Dr. Douglas Murray (Keio University, Japan) opened the second session on application of engineering in quantitative biology. By presenting novel wet systems with two or three oscillators, Dr. Yannik Rondelez (CNRS-University of Tokyo) showed the origin of direct and hidden coupling of network interactions and how the layer of hidden coupling allows the building of “winner-take-all” network.

Over an extended lunch, an interactive poster session with 29 presenters was held. Poster highlights include modeling and simulation of biochemical reactions, molecular crowding, single molecule imaging, metabolic pathway analysis, chromatin dynamics, quantitative measurement of RNA, micro-fluidic devices and bioimage informatics. Engaging discussions at the poster session stressed the need for such small-scale workshops to promote cross-disciplinary interaction. Several participants acknowledged the seeding of new collaborative bridges at the poster session.

Building new ways to push the frontiers of data assimilation is pivotal for advances in quantitative biology and this was the focus of the next session. Dr. Hiromichi Nagao (Institute of Statistical Mathematics, Japan) introduced a Bayesian estimation method to systematically estimate simulation parameters with a data-driven manner, and reported its application to estimate force distribution for cytoplasmic streaming in *C. elegans* embryos. Prof. Antonio Celani (Institut Pasteur, France) described how the complex problem of sensory response in bacterial chemotaxis can be tackled both at a mechanistic level of signal transduction from receptor to motor and at a behavioral level as response to the molecular response using Bayesian inference. Dr. Timothy Stasevich (Osaka University, Japan) elucidated model parameters for RNA polymerase II transcription dynamics using various quantitative data obtained from FRAP (fluorescence recovery after photo bleaching) and FabLEM (Fab-based live endogenous modification labeling) in living cells.

The final session of the meeting on chromosome and spindle dynamics started with Prof. Tomoyuki Tanaka's (University of Dundee, UK) presentation of ingenious experiments demonstrating the steps through which a microtubule captures chromosome. This was followed by a tour-de-force presentation of Shugoshin's role in monitoring centromeric tension and preventing errors in chromosome-microtubule attachment by Prof. Yoshinori Watanabe (University of Tokyo, Japan). Dr. Akatsuki Kimura (National Institute of Genetics, Japan) quantified and formulated the relationship between spindle length and width in *C. elegans*. Dr. Viji Draviam (University of Cambridge, UK) presented a collaborative work with the Funahashi group (Keio University, Japan) on the importance of astral microtubule pause frequency in translating cell shape cues into spindle rotating forces during human cell division.

The meeting ended with final remarks by Prof. Madan Rao who commented on the success of the day's meeting in building new interactions and also stressed on the need for similar future meetings to promote interaction between researchers in Asian and European countries.

**Figure 1:**
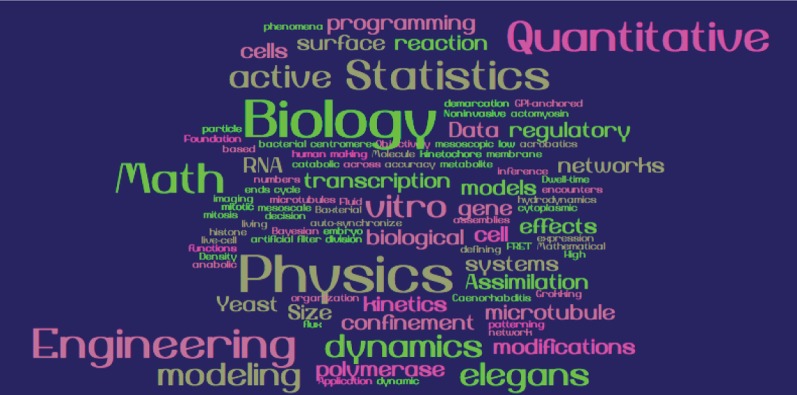
**Visual representation of keywords provided by meeting participants to describe their research area of interest.** The weighted list was generated using Word Cloud.

Researchers with a wide range of expertise had contributed to the success of the day (Figure 1). Participants were from diverse geographical areas: Japan (41%), India (12%), France (12%), China (6%), Turkey (6%), USA (6%), UK (6%), Italy (6%), Germany (1%), and also diverse disciplines: Biophysics (29%), Bioengineering (17%), Applied Mathematics (24%), Molecular Biology (24%), and Bioinformatics (6%). This meeting was made possible by funds from Royal Society, UK, Transdisciplinary Research Integration Center (TRIC) of Research Organization of Information and Systems (ROIS), Japan and Research Group on Engineering in Medicine and Biology, Institute of Industrial Science, the University of Tokyo, Japan.
